# 4-[(4-Diethyl­amino-2-hydroxy­benzyl­idene)ammonio]-3-methyl­benzene­sulfonate

**DOI:** 10.1107/S1600536809043098

**Published:** 2009-10-23

**Authors:** Xi-Shi Tai, Zeng-Bing Zhao

**Affiliations:** a Department of Chemistry and Chemical Engineering, Weifang University, Weifang 261061, People’s Republic of China; b Department of Chemistry, Qinghai Normal University, Xining 810008, People’s Republic of China

## Abstract

In the zwitterionic title compound, C_18_H_22_N_2_O_4_S, the dihedral angle between the aromatic rings is 16.39 (11)° and an intra­molecular N—H⋯O hydrogen bond occurs. In the crystal, mol­ecules are linked by O—H⋯O hydrogen bonds, forming chains propagating in [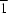
01].

## Related literature

For background to Schiff bases, see: Bu *et al.* (2001 ▶); Ranford *et al.* (1998 ▶).
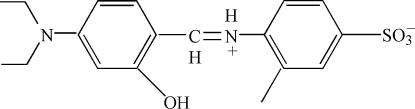


## Experimental

### 

#### Crystal data

C_18_H_22_N_2_O_4_S
*M* *_r_* = 362.44
Monoclinic, 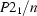

*a* = 8.230 (9) Å
*b* = 12.143 (14) Å
*c* = 18.96 (2) Å
β = 96.848 (19)°
*V* = 1882 (4) Å^3^
*Z* = 4
Mo *K*α radiation
μ = 0.20 mm^−1^
*T* = 273 K
0.18 × 0.16 × 0.12 mm

#### Data collection

Bruker SMART CCD diffractometer
Absorption correction: none
9430 measured reflections
3337 independent reflections
2737 reflections with *I* > 2σ(*I*)
*R*_int_ = 0.028

#### Refinement

*R*[*F* ^2^ > 2σ(*F* ^2^)] = 0.046
*wR*(*F* ^2^) = 0.136
*S* = 1.05
3337 reflections
227 parameters
H-atom parameters constrained
Δρ_max_ = 0.44 e Å^−3^
Δρ_min_ = −0.24 e Å^−3^

### 

Data collection: *SMART* (Bruker, 2001 ▶); cell refinement: *SAINT* (Bruker, 2001 ▶); data reduction: *SAINT*; program(s) used to solve structure: *SHELXS97* (Sheldrick, 2008 ▶); program(s) used to refine structure: *SHELXL97* (Sheldrick, 2008 ▶); molecular graphics: *SHELXTL* (Sheldrick, 2008 ▶); software used to prepare material for publication: *SHELXTL*.

## Supplementary Material

Crystal structure: contains datablocks global, I. DOI: 10.1107/S1600536809043098/hb5148sup1.cif

Structure factors: contains datablocks I. DOI: 10.1107/S1600536809043098/hb5148Isup2.hkl

Additional supplementary materials:  crystallographic information; 3D view; checkCIF report

## Figures and Tables

**Table 1:** Hydrogen-bond geometry (Å, °)

*D*—H⋯*A*	*D*—H	H⋯*A*	*D*⋯*A*	*D*—H⋯*A*
N1—H1⋯O4	0.86	1.99	2.668 (4)	135
O4—H4⋯O3^i^	0.82	1.84	2.623 (4)	160

Symmetry code: (i) 
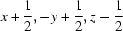
.

**Fig. 1.**
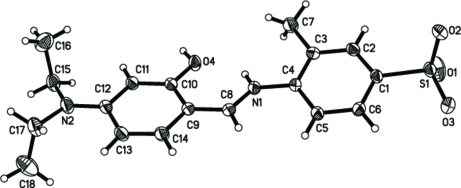
The molecular structure of (I) showing 30% displacement ellipsoids.

## supplementary crystallographic information

### Comment

Schiff bases play an important role in the field of bioinorganic chemistry because they have remarkable wide biological and pharmacological activities, such as antitumor, antidiabetic, antitubercular activities [Ranford, *et al.*, 1998; Bu, *et al.*, 2001]. In this paper, we report on the synthesis and crystal structure of the title compound, (I), (Scheme I).

The dihedral angle between the aromatic ring planes is 16.4, showing that the whole compound is not a plane molecule. The bond distances of C8—N1(1.335 (3)), S1—O1 (1.461 (3)) and S1—O2 (1.464 (2)) are consistent with the carbon-nitrogen and sulfur-oxygen double-bond lengths, respectively. In the crystal packing, the molecules form a one-dimensional chain structure by hydrogen bonds.

### Experimental

A solution of 1.0 mmol 4-(Diethylamino)salicylaldehyde was added to a solution of 1.0 mmol 3-methyl-benzenesulfonic acid in 5 ml ethanol at room temperature. The mixture was refluxed for 4 h with stirring, then the resulting precipitate was filtered, washed, and dried *in vacuo* over P_4_O_10_ for 48 h.  Brown blocks of (I) were obtained by slowly evaporating from methanol at room temperature.

### Crystal data

**Table:**

C_18_H_22_N_2_O_4_S	*F*(000) = 768
*M**_r_* = 362.44	*D*_x_ = 1.279 Mg m^−^^3^
Monoclinic, *P*2_1_/*n*	Mo *K*α radiation, λ = 0.71073 Å
Hall symbol: -P 2yn	Cell parameters from 3420 reflections
*a* = 8.230 (9) Å	θ = 2.6–27.8°
*b* = 12.143 (14) Å	µ = 0.20 mm^−^^1^
*c* = 18.96 (2) Å	*T* = 273 K
β = 96.848 (19)°	Block, brown
*V* = 1882 (4) Å^3^	0.18 × 0.16 × 0.12 mm
*Z* = 4	

### Data collection

**Table:**

Bruker SMART CCD diffractometer	2737 reflections with *I* > 2σ(*I*)
Radiation source: fine-focus sealed tube	*R*_int_ = 0.028
graphite	θ_max_ = 25.1°, θ_min_ = 2.0°
ω scans	*h* = −9→9
9430 measured reflections	*k* = −13→14
3337 independent reflections	*l* = −19→22

### Refinement

**Table:**

Refinement on *F*^2^	Primary atom site location: structure-invariant direct methods
Least-squares matrix: full	Secondary atom site location: difference Fourier map
*R*[*F*^2^ > 2σ(*F*^2^)] = 0.046	Hydrogen site location: inferred from neighbouring sites
*wR*(*F*^2^) = 0.136	H-atom parameters constrained
*S* = 1.05	*w* = 1/[σ^2^(*F*_o_^2^) + (0.0682*P*)^2^ + 0.8828*P*] where *P* = (*F*_o_^2^ + 2*F*_c_^2^)/3
3337 reflections	(Δ/σ)_max_ < 0.001
227 parameters	Δρ_max_ = 0.44 e Å^−^^3^
0 restraints	Δρ_min_ = −0.24 e Å^−^^3^

### Special details

**Table:**

Geometry. All e.s.d.'s (except the e.s.d. in the dihedral angle between two l.s. planes) are estimated using the full covariance matrix. The cell e.s.d.'s are taken into account individually in the estimation of e.s.d.'s in distances, angles and torsion angles; correlations between e.s.d.'s in cell parameters are only used when they are defined by crystal symmetry. An approximate (isotropic) treatment of cell e.s.d.'s is used for estimating e.s.d.'s involving l.s. planes.
Refinement. Refinement of *F*^2^ against ALL reflections. The weighted *R*-factor *wR* and goodness of fit *S* are based on *F*^2^, conventional *R*-factors *R* are based on *F*, with *F* set to zero for negative *F*^2^. The threshold expression of *F*^2^ > σ(*F*^2^) is used only for calculating *R*-factors(gt) *etc*. and is not relevant to the choice of reflections for refinement. *R*-factors based on *F*^2^ are statistically about twice as large as those based on *F*, and *R*- factors based on ALL data will be even larger.

### Fractional atomic coordinates and isotropic or equivalent isotropic displacement parameters (Å^2^)

**Table:**

	*x*	*y*	*z*	*U*_iso_*/*U*_eq_	
S1	0.24693 (7)	0.19093 (5)	0.55953 (3)	0.0423 (2)	
O1	0.2152 (3)	0.30892 (16)	0.56444 (12)	0.0792 (7)	
O2	0.1087 (2)	0.12620 (16)	0.52607 (9)	0.0582 (5)	
O3	0.3181 (2)	0.14533 (16)	0.62907 (8)	0.0561 (5)	
O4	0.8678 (2)	0.22390 (14)	0.23964 (8)	0.0496 (4)	
H4	0.8646	0.2536	0.2006	0.074*	
N1	0.7742 (2)	0.15936 (16)	0.36312 (9)	0.0394 (4)	
H1	0.7599	0.2026	0.3270	0.047*	
N2	1.3356 (2)	0.04143 (18)	0.15486 (10)	0.0513 (5)	
C1	0.4078 (3)	0.17934 (17)	0.50261 (11)	0.0359 (5)	
C2	0.3808 (3)	0.22288 (18)	0.43351 (11)	0.0396 (5)	
H2	0.2808	0.2558	0.4183	0.047*	
C3	0.5015 (3)	0.21780 (18)	0.38679 (11)	0.0373 (5)	
C4	0.6527 (3)	0.16570 (17)	0.41184 (11)	0.0349 (5)	
C5	0.6818 (3)	0.1232 (2)	0.48125 (11)	0.0447 (6)	
H5	0.7821	0.0911	0.4971	0.054*	
C6	0.5588 (3)	0.1295 (2)	0.52658 (11)	0.0437 (6)	
H6	0.5771	0.1008	0.5723	0.052*	
C7	0.4692 (3)	0.2677 (2)	0.31182 (13)	0.0572 (7)	
H7A	0.3545	0.2828	0.3008	0.086*	
H7B	0.5030	0.2165	0.2778	0.086*	
H7C	0.5300	0.3349	0.3101	0.086*	
C8	0.9062 (3)	0.09465 (19)	0.36714 (11)	0.0394 (5)	
H8	0.9291	0.0510	0.4074	0.047*	
C9	1.0147 (3)	0.08791 (19)	0.31374 (11)	0.0381 (5)	
C10	0.9949 (3)	0.15006 (18)	0.24798 (11)	0.0368 (5)	
C11	1.0999 (3)	0.13405 (19)	0.19596 (11)	0.0410 (5)	
H11	1.0830	0.1739	0.1539	0.049*	
C12	1.2333 (3)	0.05717 (19)	0.20639 (11)	0.0408 (5)	
C13	1.2563 (3)	−0.0028 (2)	0.27321 (12)	0.0456 (6)	
H13	1.3434	−0.0516	0.2823	0.055*	
C14	1.1492 (3)	0.0123 (2)	0.32326 (12)	0.0452 (6)	
H14	1.1651	−0.0285	0.3650	0.054*	
C15	1.3247 (3)	0.1109 (2)	0.08923 (13)	0.0555 (7)	
H15A	1.4317	0.1149	0.0728	0.067*	
H15B	1.2924	0.1850	0.1006	0.067*	
C16	1.2015 (5)	0.0648 (3)	0.02963 (16)	0.0824 (10)	
H16A	1.2287	−0.0102	0.0204	0.124*	
H16B	1.2051	0.1079	−0.0126	0.124*	
H16C	1.0934	0.0680	0.0438	0.124*	
C17	1.4625 (3)	−0.0487 (2)	0.15911 (15)	0.0596 (7)	
H17A	1.4728	−0.0754	0.1116	0.072*	
H17B	1.4268	−0.1096	0.1865	0.072*	
C18	1.6273 (4)	−0.0090 (3)	0.1931 (2)	0.0841 (10)	
H18A	1.6586	0.0554	0.1687	0.126*	
H18B	1.7073	−0.0659	0.1902	0.126*	
H18C	1.6209	0.0086	0.2420	0.126*	

### Atomic displacement parameters (Å^2^)

**Table:**

	*U*^11^	*U*^22^	*U*^33^	*U*^12^	*U*^13^	*U*^23^
S1	0.0342 (3)	0.0489 (4)	0.0459 (3)	0.0009 (2)	0.0136 (2)	−0.0093 (2)
O1	0.0831 (15)	0.0529 (12)	0.1121 (17)	0.0114 (10)	0.0548 (13)	−0.0103 (11)
O2	0.0361 (9)	0.0829 (13)	0.0558 (10)	−0.0121 (9)	0.0062 (8)	−0.0059 (9)
O3	0.0521 (11)	0.0770 (12)	0.0408 (9)	0.0002 (9)	0.0121 (8)	−0.0073 (8)
O4	0.0489 (10)	0.0619 (10)	0.0405 (9)	0.0212 (8)	0.0156 (7)	0.0156 (8)
N1	0.0357 (10)	0.0501 (11)	0.0333 (9)	0.0066 (8)	0.0074 (7)	0.0048 (8)
N2	0.0420 (11)	0.0646 (13)	0.0501 (12)	0.0110 (10)	0.0170 (9)	0.0091 (10)
C1	0.0314 (11)	0.0381 (11)	0.0391 (11)	0.0000 (9)	0.0082 (9)	−0.0078 (9)
C2	0.0312 (11)	0.0421 (12)	0.0448 (12)	0.0058 (9)	0.0022 (9)	−0.0017 (10)
C3	0.0343 (11)	0.0395 (11)	0.0374 (11)	0.0018 (9)	0.0012 (9)	0.0011 (9)
C4	0.0328 (11)	0.0401 (12)	0.0325 (11)	0.0004 (9)	0.0065 (8)	−0.0028 (9)
C5	0.0344 (12)	0.0636 (15)	0.0364 (12)	0.0158 (11)	0.0057 (9)	0.0036 (10)
C6	0.0406 (13)	0.0595 (15)	0.0315 (11)	0.0107 (11)	0.0059 (9)	0.0037 (10)
C7	0.0469 (15)	0.0750 (18)	0.0496 (14)	0.0103 (13)	0.0048 (11)	0.0190 (13)
C8	0.0358 (12)	0.0511 (13)	0.0309 (11)	0.0024 (10)	0.0026 (9)	0.0037 (9)
C9	0.0336 (11)	0.0486 (13)	0.0320 (11)	0.0029 (10)	0.0038 (9)	0.0018 (9)
C10	0.0321 (11)	0.0426 (12)	0.0357 (11)	0.0030 (9)	0.0041 (9)	0.0018 (9)
C11	0.0381 (12)	0.0505 (13)	0.0352 (11)	0.0037 (10)	0.0077 (9)	0.0067 (10)
C12	0.0323 (11)	0.0508 (13)	0.0403 (12)	0.0019 (10)	0.0082 (9)	0.0007 (10)
C13	0.0349 (12)	0.0543 (14)	0.0480 (13)	0.0110 (11)	0.0063 (10)	0.0074 (11)
C14	0.0402 (13)	0.0569 (14)	0.0387 (12)	0.0088 (11)	0.0054 (10)	0.0118 (10)
C15	0.0516 (15)	0.0636 (16)	0.0552 (15)	0.0077 (13)	0.0231 (12)	0.0119 (12)
C16	0.104 (3)	0.084 (2)	0.0573 (18)	0.019 (2)	0.0021 (17)	−0.0009 (16)
C17	0.0518 (16)	0.0632 (17)	0.0674 (17)	0.0093 (13)	0.0218 (13)	0.0080 (13)
C18	0.0546 (18)	0.076 (2)	0.122 (3)	−0.0037 (16)	0.0109 (18)	0.021 (2)

### Geometric parameters (Å, °)

**Table:**

S1—O1	1.461 (3)	C7—H7C	0.9600
S1—O2	1.464 (2)	C8—C9	1.430 (3)
S1—O3	1.485 (2)	C8—H8	0.9300
S1—C1	1.811 (3)	C9—C14	1.433 (3)
O4—C10	1.372 (3)	C9—C10	1.450 (3)
O4—H4	0.8200	C10—C11	1.401 (3)
N1—C8	1.335 (3)	C11—C12	1.437 (3)
N1—C4	1.442 (3)	C11—H11	0.9300
N1—H1	0.8600	C12—C13	1.454 (3)
N2—C12	1.377 (3)	C13—C14	1.383 (3)
N2—C15	1.497 (3)	C13—H13	0.9300
N2—C17	1.508 (3)	C14—H14	0.9300
C1—C2	1.406 (3)	C15—C16	1.531 (4)
C1—C6	1.408 (3)	C15—H15A	0.9700
C2—C3	1.409 (3)	C15—H15B	0.9700
C2—H2	0.9300	C16—H16A	0.9600
C3—C4	1.426 (3)	C16—H16B	0.9600
C3—C7	1.539 (3)	C16—H16C	0.9600
C4—C5	1.407 (3)	C17—C18	1.509 (4)
C5—C6	1.406 (3)	C17—H17A	0.9700
C5—H5	0.9300	C17—H17B	0.9700
C6—H6	0.9300	C18—H18A	0.9600
C7—H7A	0.9600	C18—H18B	0.9600
C7—H7B	0.9600	C18—H18C	0.9600
			
O1—S1—O2	114.73 (14)	C8—C9—C10	124.3 (2)
O1—S1—O3	111.33 (13)	C14—C9—C10	116.60 (19)
O2—S1—O3	112.88 (12)	O4—C10—C11	122.38 (19)
O1—S1—C1	105.25 (11)	O4—C10—C9	116.42 (18)
O2—S1—C1	106.60 (12)	C11—C10—C9	121.2 (2)
O3—S1—C1	105.14 (12)	C10—C11—C12	121.3 (2)
C10—O4—H4	109.5	C10—C11—H11	119.4
C8—N1—C4	128.05 (19)	C12—C11—H11	119.4
C8—N1—H1	116.0	N2—C12—C11	121.0 (2)
C4—N1—H1	116.0	N2—C12—C13	121.4 (2)
C12—N2—C15	122.0 (2)	C11—C12—C13	117.61 (19)
C12—N2—C17	122.8 (2)	C14—C13—C12	120.3 (2)
C15—N2—C17	115.20 (19)	C14—C13—H13	119.9
C2—C1—C6	119.88 (19)	C12—C13—H13	119.9
C2—C1—S1	118.72 (17)	C13—C14—C9	123.0 (2)
C6—C1—S1	121.39 (18)	C13—C14—H14	118.5
C1—C2—C3	121.6 (2)	C9—C14—H14	118.5
C1—C2—H2	119.2	N2—C15—C16	112.1 (2)
C3—C2—H2	119.2	N2—C15—H15A	109.2
C2—C3—C4	117.6 (2)	C16—C15—H15A	109.2
C2—C3—C7	120.4 (2)	N2—C15—H15B	109.2
C4—C3—C7	122.0 (2)	C16—C15—H15B	109.2
C5—C4—C3	121.26 (19)	H15A—C15—H15B	107.9
C5—C4—N1	121.70 (19)	C15—C16—H16A	109.5
C3—C4—N1	117.04 (19)	C15—C16—H16B	109.5
C6—C5—C4	119.8 (2)	H16A—C16—H16B	109.5
C6—C5—H5	120.1	C15—C16—H16C	109.5
C4—C5—H5	120.1	H16A—C16—H16C	109.5
C5—C6—C1	119.9 (2)	H16B—C16—H16C	109.5
C5—C6—H6	120.1	N2—C17—C18	111.9 (3)
C1—C6—H6	120.1	N2—C17—H17A	109.2
C3—C7—H7A	109.5	C18—C17—H17A	109.2
C3—C7—H7B	109.5	N2—C17—H17B	109.2
H7A—C7—H7B	109.5	C18—C17—H17B	109.2
C3—C7—H7C	109.5	H17A—C17—H17B	107.9
H7A—C7—H7C	109.5	C17—C18—H18A	109.5
H7B—C7—H7C	109.5	C17—C18—H18B	109.5
N1—C8—C9	124.4 (2)	H18A—C18—H18B	109.5
N1—C8—H8	117.8	C17—C18—H18C	109.5
C9—C8—H8	117.8	H18A—C18—H18C	109.5
C8—C9—C14	119.0 (2)	H18B—C18—H18C	109.5
			
O1—S1—C1—C2	−58.2 (2)	N1—C8—C9—C10	−0.4 (4)
O2—S1—C1—C2	64.0 (2)	C8—C9—C10—O4	3.7 (3)
O3—S1—C1—C2	−175.90 (17)	C14—C9—C10—O4	−178.8 (2)
O1—S1—C1—C6	120.8 (2)	C8—C9—C10—C11	−175.8 (2)
O2—S1—C1—C6	−117.0 (2)	C14—C9—C10—C11	1.7 (3)
O3—S1—C1—C6	3.1 (2)	O4—C10—C11—C12	179.2 (2)
C6—C1—C2—C3	0.2 (3)	C9—C10—C11—C12	−1.3 (3)
S1—C1—C2—C3	179.24 (17)	C15—N2—C12—C11	6.4 (4)
C1—C2—C3—C4	0.6 (3)	C17—N2—C12—C11	−171.6 (2)
C1—C2—C3—C7	−179.1 (2)	C15—N2—C12—C13	−173.4 (2)
C2—C3—C4—C5	−1.5 (3)	C17—N2—C12—C13	8.6 (4)
C7—C3—C4—C5	178.2 (2)	C10—C11—C12—N2	179.7 (2)
C2—C3—C4—N1	178.98 (19)	C10—C11—C12—C13	−0.5 (3)
C7—C3—C4—N1	−1.3 (3)	N2—C12—C13—C14	−178.2 (2)
C8—N1—C4—C5	17.3 (3)	C11—C12—C13—C14	1.9 (3)
C8—N1—C4—C3	−163.2 (2)	C12—C13—C14—C9	−1.6 (4)
C3—C4—C5—C6	1.5 (3)	C8—C9—C14—C13	177.4 (2)
N1—C4—C5—C6	−178.9 (2)	C10—C9—C14—C13	−0.3 (3)
C4—C5—C6—C1	−0.7 (4)	C12—N2—C15—C16	−87.6 (3)
C2—C1—C6—C5	−0.2 (3)	C17—N2—C15—C16	90.5 (3)
S1—C1—C6—C5	−179.17 (18)	C12—N2—C17—C18	−93.3 (3)
C4—N1—C8—C9	175.3 (2)	C15—N2—C17—C18	88.5 (3)
N1—C8—C9—C14	−177.9 (2)		

### Hydrogen-bond geometry (Å, °)

**Table:**

*D*—H···*A*	*D*—H	H···*A*	*D*···*A*	*D*—H···*A*
N1—H1···O4	0.86	1.99	2.668 (4)	135
O4—H4···O3^i^	0.82	1.84	2.623 (4)	160

Symmetry codes: (i) *x*+1/2, −*y*+1/2, *z*−1/2.
